# Right hemisphere damage: Communication processing in adults evaluated by the Brazilian Protocole MEC – Bateria MAC

**DOI:** 10.1590/S1980-57642008DN10300008

**Published:** 2007

**Authors:** Rochele Paz Fonseca, Jandyra Maria Guimarães Fachel, Márcia Lorena Fagundes Chaves, Francéia Veiga Liedtke, Maria Alice de Mattos Pimenta Parente

**Affiliations:** 1 PhD in Developmental Psychology, Institute of Psychology, Federal University of Rio Grande do Sul (UFRGS). Laboratory of Neuropsycholinguistics, Institute of Psychology, Federal University of Rio Grande do Sul (UFRGS), Brazil.; 2 PhD in Statistics, University of London. Department of Statistics, Institute of Mathematics, Federal University of Rio Grande do Sul (UFRGS), Brazil.; 3 PhD in Medicine, Federal University of Rio Grande do Sul (UFRGS). Department of Internal Medicine, Faculty of Medicine, Federal University of Rio Grande do Sul (UFRGS) and Neurology Service of the Hospital of Clinics of Porto Alegre (HCPA), Brazil.; 4 Psychology Undergraduate, Federal University of Rio Grande do Sul (UFRGS). Laboratory of Neuropsycholinguistics, Institute of Psychology, Federal University of Rio Grande do Sul (UFRGS), Brazil.; 5 PhD in Psychology, University of São Paulo (USP). Graduate Program in Psychology, Institute of Psychology, Department of Developmental and Personality Psychology, Federal University of Rio Grande do Sul (UFRGS), Porto Alegre, Brazil.

**Keywords:** neuropsychology, right hemisphere, brain damage, communication

## Abstract

Right-brain-damaged individuals may present discursive, pragmatic, lexical-semantic and/or prosodic disorders.

**Objective:**

To verify the effect of right hemisphere damage on communication processing evaluated by the Brazilian version of the Protocole Montréal d’Évaluation de la Communication (Montreal Communication Evaluation Battery) – Bateria Montreal de Avaliação da Comunicação, Bateria MAC, in Portuguese.

**Methods:**

A clinical group of 29 right-brain-damaged participants and a control group of 58 non-brain-damaged adults formed the sample. A questionnaire on sociocultural and health aspects, together with the Brazilian MAC Battery was administered.

**Results:**

Significant differences between the clinical and control groups were observed in the following MAC Battery tasks: conversational discourse, unconstrained, semantic and orthographic verbal fluency, linguistic prosody repetition, emotional prosody comprehension, repetition and production. Moreover, the clinical group was less homogeneous than the control group.

**Conclusions:**

A right-brain-damage effect was identified directly, on three communication processes: discursive, lexical-semantic and prosodic processes, and indirectly, on pragmatic process.

## Abstract (pt)

Os indivíduos com lesão vascular de hemisfério direito podem apresentar distúrbios discursivos, pragmáticos, léxico-semânticos e/ou prosódicos.

**Objetivo:**

Verificar o efeito da lesão de hemisfério direito no processamento comunicativo avaliado pela versão Brasileira do Protocole Montréal d’Évaluation de la Communication – Bateria Montreal de Avaliação da Comunicação, Bateria MAC (em português).

**Métodos:**

Participaram do estudo um grupo clínico, com 29 adultos lesados de hemisfério direito, e um grupo controle, com 58 adultos sem lesão neurológica. Foram administrados um questionário de dados socioculturais e aspectos da saúde e a Bateria MAC.

**Resultados:**

Houve diferenças significativas entre os grupos clínico e controle nas tarefas da Bateria MAC discurso conversacional, evocação lexical livre, com critério ortográfico e semântico, prosódia lingüística repetição, prosódia emocional compreensão, repetição e produção. Além disso, o grupo clínico mostrou-se menos homogêneo do que o controle.

**Conclusões:**

Identificou-se o efeito da lesão de hemisfério direito em três processamentos comunicativos: discursivo, léxico-semântico e prosódico e, indiretamente, no processamento pragmático.

Right hemisphere (RH) damage was first associated to linguistic disorders less than 50 years ago.^1,2^ Moreover, only in the past two decades, systematic studies on such communication deficits have been done.^3^ Thus, differently from the association between the left hemisphere (LH) and linguistic abilities known since 1861, the literature has just recently considered the role of the damaged RH in linguistic disorders.^4,5^ This role has been studied in many different manners. This study focuses on the RH damage and its effects on communication. Since the late 1980s, the relationship between the RH and communication has been highlighted in studies developed with neurologically preserved individuals as well as with individuals presenting a lesion to this side of the brain, both in behavioral and neuroimaging tasks.^4,6-9^ Thus, communicative disorders following an RH lesion have been increasingly described in the literature, encompassing discursive abilities,^10^ pragmatic-inferential,^11^ lexical-semantic^12^ and prosodic disorders.^13^

However, few studies in the international literature have simultaneously investigated the four communicative components – discursive, pragmatic, lexical-semantic and prosodic – which are possibly impaired following an RH lesion. In general, each is investigated separately. This reduced amount of investigation is probably related to the fewer instruments available to systematically evaluate communicative processing linked to the RH. In this context, only two studies have examined the four components in samples of right hemisphere-brain-damaged (RHBD) subjects. Each study used a different evaluation tool: one study employed the Right Hemisphere Communication Battery,^14,15^ and the other used the Protocole Montréal d’Évaluation de la Communication – Protocole MEC.^1,3^ Besides these two studies, the Right Hemisphere Language Battery has also been used to assess the pragmatic, lexical-semantic and discursive components.^16,17^ In a complementary manner, the communicative deficit profiles of RHBD patients had previously been characterized by an evaluation of the discursive and lexical-semantic components.^18^ Thus, there is evidence to suggest that more studies are needed in order to characterize the communicative deficits in the RHBD population. It is important to consider that such investigations of clinical description depend on specific instruments for the assessment of the possible communicative deficits presented by the target neurological population.

From a Brazilian perspective, only one empirical study analyzing a sample of RHBD individuals has been published.^19^ However, in this research only the pragmatic component had been investigated. Therefore, to the best of our knowledge, the present study is a pioneer investigation in Brazil verifying the effect of RH damage in a group with lesion on this side of the brain, by simultaneously evaluating the four communicative components affected in this population: discursive, pragmatic, lexical-semantic and prosodic components. To achieve this the evaluation of communicative processing has been based on the Montreal Communication Evaluation Battery diagnostic tool – MAC Battery (in Portuguese, Bateria Montreal de Avaliação da Comunicação – Bateria MAC),^20^ which corresponds to the Brazilian version of the Protocole MEC,^1^ the first instrument adapted to Brazilian Portuguese for examining communication following RH brain damage.^21^ Two factors have probably led to this gap in the Brazilian literature:

1) the lack of specific instruments to evaluate disturbances linked to the RH adequately adapted to the Brazilian social, linguistic and cultural setting; and
2) low dissemination in Brazil of knowledge regarding the Right Hemisphere Syndrome – a set of cognitive, communicative and behavioral signs and symptoms following a neurological disorder in the RH.

In a bid to increase propagation of knowledge on the Right Hemisphere Syndrome in the international context, studies investigating simultaneously the four communicative processes potentially affected by an RH lesion have provided a clinical characterization of the communicative deficits. Four subgroups of RHBD individuals were identified according to their similarities in terms of communicative performance:

1) a subgroup with discursive, pragmatic, lexical-semantic and prosodic disorders, characterized by a limited recall of stories, difficulties in adapting to the interlocutor, reduced comprehension of non-literal language, diminished verbal fluency and deficit in intonational expression;
2) a subgroup with impaired pragmatic, lexical-semantic and prosodic abilities;
3) a subgroup with changes only in lexical-semantic processing; and,
4) a subgroup with no communicative deficits.^3^

In a study comparing a group of RHBD individuals and a group of LH brain-damaged individuals to a control group, significant differences between the clinical groups were not found, only between RHBD and the control group and between LH brain-damaged group and the control participants. These differences were found, in tasks evaluating humor, emotional prosody, indirect speech acts, metaphors, inferences, sarcasm and ambiguous meanings and discourse.^15^ However, according to observations, not all RHBD individuals present communication processing impairments. Although there are no epidemiologic studies on the prevalence of this type of processing in RHBD individuals, some estimates have been drawn suggesting that some 50% of RHBD individuals present communicative disturbances.^3,22,23^ Moreover, there is no homogeneity in the manifestation of these disturbances in the neuropsychopathological population referred to here.^6^

The current investigation intended to answer the following questions, with the aim of verifying the effect of RH damage on communicative processing in individuals with this neurological disorder compared to a control group:

1) Are there quantitative differences between the communicative performance of RHBD individuals and non-damaged individuals in the four communicative processes evaluated by the Brazilian MAC Battery?
2) Do the groups under investigation differ regarding the homogeneity of their communicative performance?

Two hypotheses have been formulated as an attempt to answer these questions:

1) Significant differences will be found between RHBD adults and non-brain-damaged adults in the four communicative processes examined by the Brazilian MAC Battery, and the differences in the lexical-semantic component will be less significant compared to the other components; and
2) The RHBD group will be less homogeneous regarding their performance than the non-brain-damaged group.

## Methods

### Participants

The sample investigated in this study comprised two groups:

1) a clinical group: 29 RHBD adults, and
2) a control group: 58 adults with no neurological lesions.

The descriptive data of the sample of the two groups regarding age, schooling, reading and writing habits frequency, as well as the distribution by gender, are shown in Table 1. It is important to state that, based on Student’s t-Test, there were no statistically significant differences between the groups regarding the variables of age, schooling, reading and writing habits frequency. The proportion between male and female participants has been shown to differ in the Chi-square Test (p≤001). However, no statistically significant differences were found in the scores of the Brazilian MAC Battery subtests for male and female participants in both groups according to the Student’s t-Test. Regarding manual dominance, in the clinical group all participants were right-handed, while in the control group, there were two left-handed individuals. The handedness was self-reported.

**Table 1:** Descriptive data of the groups regarding age, schooling, frequency of written language habits and gender distribution

	Groups	
Descriptive data	Right-brain-damaged Non-brain-damaged	
Age M* (SD^†^)	58.34 (13.12)	57.71 (12.52)
Schooling M (SD)	8.52 (5.89)	9.41 (6.42)
Written language habits frequency score M (SD)		
Gender (Female/Male)	14/15	45/13

* M, stands for mean;

† SD, standard deviation.

The clinical group sample size has been defined upon the application of a sample calculation (WINPEPI, module compare 2, version 1.47). A level of significance of 0.05, a power of 90% and a reason 2:1 (controls:case) have been considered, in order to detect a clinically relevant difference of two standard deviations. A difference of 1.5 or of two standard deviations has been observed in two studies in which the communicative performance of neurologically preserved participants and of RHBD individuals has been compared.^3,15^ The minimal sample size stipulated for the clinical group was 9 participants and, for the control group, 18 individuals.

The participants of the clinical group have been selected through the sampling technique of non-random convenience from neurological ambulatory service records at public and private hospitals in the region of Porto Alegre, RS. The inclusion criteria were as follows: RH lesion diagnosed by neuroimaging techniques and neurological assessment (18 participants underwent computerized tomography, and 11 tomography and magnetic resonance imaging); ischemic (25 participants) or hemorrhagic vascular accident (4 participants); no occurrence of pre-frontal lesion to avoid executive dysfunction and behavioral disorders usually referred in patients with this lesion site (the distribution of the clinical group participants regarding the RH lesion sites is presented in Table 2); minimal time of three weeks post-onset (average of 16.66 months, standard deviation 23.62); absence of any other type of neurological impairment, such as tumors, traumatic brain injury; right hand dominance; and, no participation in speech therapy and/or neuropsychological rehabilitation programs. Participants of the clinical group with a first and single RH vascular lesion were preferred (only one participant had two vascular accidents, both in the RH). When it comes to hemineglect occurrence, 07 clinical participants presented signs of this syndrome in a screening cancellation lines task. They were included because visual stimuli of Brazilian MAC Battery were also audio presented. Besides this, the examiner guided these patients’ vision pointing from the beginning until the end of each word or sentence.

**Table 2:** Distribution of the clinical group participants regarding right hemisphere lesion sites.

Lesion sites	Number of right-brain- damaged participants
Frontal and parietal cortex	6
Subcortical zones (periventricular, perinsular, basal ganglia)	6
Temporal and parietal cortex	5
Frontal cortex and basal ganglia	3
Temboral cortex and basal ganglia	2
Frontal cortex	2
Parietal cortex	2
Frontal, temporal and parietal cortex and basal ganglia	2
Frontal and parietal cortex and thalamus	1
Total	29

Regarding the participants of the control group, the majority were selected from the original normalization data set of the Brazilian MAC Battery instrument.^20^ Two control individuals were chosen for each case of the clinical group and matched for age, schooling and reading and writing habits frequency scores (2:1 design). Moreover, the following inclusion criteria were common to the two groups: no existing conditions of dementia (Mini-Mental^27^ score, adapted for the local Brazilian population,^25^ ≥24 points, for individuals with more than 4 years of school education, and 17, for participants with ≤4 years of school education); no existing conditions of depression (evaluated through the Brazilian version of Geriatric Depression Scale);^26^ no existing conditions of current or previous history of psychoactive substance use nor alcohol abuse, psychiatric, neurological and/or sensorial (non-corrected hearing and/or visual problems) disorders. These criteria were verified from the participants’ self-reports in a questionnaire on socio-cultural and health data.

### Procedures

In accordance with the ethics related to research on human beings, the participation of the individuals in the study was voluntary. Participants and relatives signed an Informed Consent. The Committee of Ethics in Research of the Hospital de Clínicas de Porto Alegre (protocol number 06283) approved the project of this study.

The instruments described in the next subsection were administered to the control group in a session of approximately one hour and thirty minutes. To the clinical group, the evaluation was done in two or three sessions, with a duration of one hour each, subject to the availability and fatigue of the participants.

All the answers given by the participants were recorded on audio equipment for posterior transcription and analysis. The same examiner, a neuropsychologist expert in language, and rigorously trained by the authors of the original Protocole MEC, analyzed the answers to ensure uniformity in the attributing of scores in accordance with the Brazilian MAC Battery Manual of Application and Interpretation.^23^ A sample of 15% of the protocols of the clinical group and of the control group, randomly selected, was analyzed by two independent specialized judges, and an agreement coefficient of over 0.80 between one of the judges and the main examiner was found in all Brazilian MAC Battery tasks.

### Instruments

#### 1) Questionnaire on socio-cultural and health aspects

The participants were instructed to answer a questionnaire that investigated issues regarding demographic, cultural and communicative data and medical history (general, sensorial and neurological health). Through this instrument, all socio-demographic and health criteria were investigated.

Regarding the frequency of writing and reading habits, scores ranging from 4 to 0 were attributed. The “every day” frequency corresponded to a score of 4, some days per week corresponded to 3, once a week, 2, rarely, 1, and never, 0. The total score of written language habits frequency was obtained through the addition of seven partial scores: reading of magazines (1), newspapers (2), books (3), others, such as emails (4), and the activity of writing texts (5), messages (6) and others, such as emails (7).

#### 2) The Brazilian MAC Battery

The Bateria Montreal de Avaliação da Comunicação – Bateria MAC (Montreal Communication Evaluation Battery)^20^, the Brazilian version of the Protocole MEC,^1^ aims to evaluate discursive, pragmatic-inferential, lexical-semantic and prosodic abilities of the communicative processing of neurological populations, mainly RHBD subjects. It is composed of 14 subtests, briefly described in Appendix A, in the order they have been administered.

### Data analysis

Data were analyzed based on inferential and descriptive statistics (Student’s t-Test for independent samples) using the SPSS package, version 12 to compare mean values obtained for the clinical and control groups. Also, the Chi-square Test was used to compare the proportion of individuals who made the expected inference in the clinical group and control group, and the distribution per score in titles 1 and 2 of the narrative discourse task.

## Results

**Figure 1:**
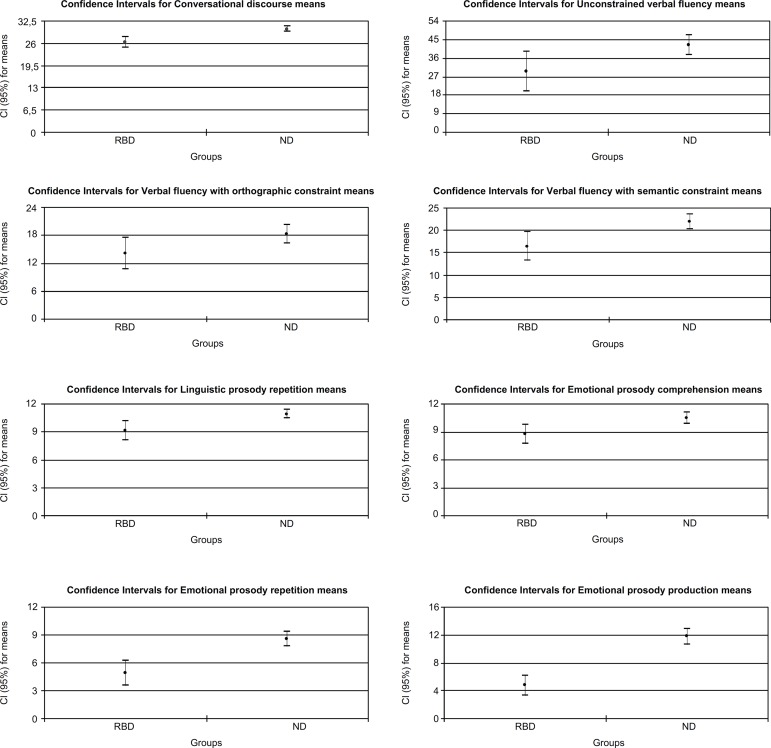
Confidence Intervals for means of the MAC Battery tasks with significant differences between groups. *CI means Confidence Intervals; RBD, right-brain-damaged participants; ND, non-damaged participants.

The study results are presented in Tables 3 and 4, and in Figure 1. Table 3 shows mean and standard deviation values obtained for the clinical and the control groups in the Brazilian MAC Battery, as well as the level of significance of the difference between the communicative performance means of the two groups through the Student’s t-Test. Regarding results obtained in the questionnaire on deficit awareness, 7 (24.0%) of the 29 RHBD participants presented deficiency in the awareness evaluated. Also, coefficients of the variation of the groups in the Brazilian MAC Battery can be seen in Table 3.

**Table 3:** Means, standard deviations and coefficients of variability of the groups on the MAC Battery.

Tasks ( /maximum score)	Groups						
	Right-brain-damaged				Non-brain-damaged		
	M^†^	SD^‡^	CV^§^		M	SD	CV
Conversational discourse ( /34)***	26.24	3.91	0.15		30.07	2.73	0.09
Metaphor interpretation ( /40)	28.10	6.74	0.24		28.93	7.16	0.26
Unconstrained verbal fluency**	29.48	25.54	0.87		42.22	17.46	0.41
Linguistic prosody comprehension ( /12)	8.34	3.10	0.37		9.45	2.37	0.25
Linguistic prosody repetition ( /12)**	9.14	2.58	0.28		10.91	1.65	0.15
Narrative discourse: partial retelling, main information ( /18)	11.07	4.14	0.37		11.47	3.88	0.34
Narrative discourse: full retelling ( /29)	6.66	3.65	0.55		7.91	3.39	0.43
Narrative discourse: comprehension questions ( /12)	9.28	2.32	0.25		9.19	2.60	0.28
Verbal fluency with orthographic constraint *	14.10	8.84	0.63		18.29	7.54	0.41
Emotional prosody comprehension** ( /12)	8.76	2.65	0.30		10.47	2.21	0.21
Emotional prosody repetition*** ( /12)	4.86	3.51	0.72		8.57	2.96	0.35
Indirect speech acts interpretation ( /40)	29.97	3.80	0.13		30.50	3.99	0.13
Verbal fluency with semantic constraint **	16.41	8.53	0.52		21.86	6.36	0.29
Emotional prosody production*** ( /18)	4.75	3.77	0.79		11.74	4.22	0.36
Semantic judgement: identification score ( /24)	21.72	2.77	0.13		22.41	2.25	0.10
Semantic judgement: explanation score ( /12)	7.24	3.03	0.42		8.24	3.19	0.39

* p≤0.05;

** p≤0.01;

*** p≤0.001;

† M, stands for mean;

‡ SD, standard deviation;

§ CV, coefficient of variability.

**Table 4:** Proportion of individuals per group in terms of presence of inferencing and scores for titles 1 and 2 in narrative discourse task

	Groups	
Category variables	Right-brain-damaged	Non-brain-damaged
Presence of inference	20 (69.0%)	44 (75.9%)
Absence of inference	9 (31.0%)	14 (24.1%)
Title 1 score 0	12 (41.4%)	9 (15.5%)
Title 1 score 1	5 (17.2%)	23 (39.7%)
Title 1 score 2	12 (41.4%)	26 (44.8%)
Title 2 score 0	9 (31.0%)	7 (12.1%)
Title 2 score 1	5 (17.2%)	20 (34.5%)
Title 2 score 2	15 (51.7%)	31 (53.4%)

The analysis of the results presented in Table 3 indicates the presence of a significant difference between the performance of the clinical group and the control group in the following Brazilian MAC Battery tasks: conversational discourse, unconstrained verbal fluency, verbal fluency with orthographic constraint, verbal fluency with semantic constraint, linguistic prosody repetition, emotional prosody comprehension, emotional prosody repetition and emotional prosody production. A general tendency for a better performance by non-brain-damaged individuals compared to RHBD participants was observed. Moreover, based on the coefficient of variation, a more reduced homogeneity in the performance of the clinical group compared to the control group was registered in majority of tasks. In the Levene test for equality of variances, variances between the groups were significantly unequal in the following subtests: conversational discourse (p≤0.05), verbal fluency with semantic constraint (p≤0.01), linguistic prosody comprehension (p≤0.05) and linguistic prosody repetition (p≤0,001).

For better visualization of the performance variability of the two groups, Figure 1 presents graphs with the confidence intervals for mean values of clinical and control groups for tasks in which a significant difference between the groups was found. This Figure shows that RHBD participants’ confidence intervals are visibly larger than those of the non-brain-damaged group. Intersection among the confidence intervals, where present, is small.

Data from narrative discourse tasks are displayed in Table 4. The table shows the proportion of RHBD and non-brain damaged individuals who have made the expected inference and who have scored 0, 1 or 2 in titles 1 and 2.

Data in Table 4 demonstrate that, regarding inferential processing, a larger proportion of non-damaged individuals elucidated the expected inference, although no significant differences among proportions in the Chi-square test were found (p=0.330). Regarding the titles given by the groups to the orally presented narrative, both titles 1 and 2 were associated to a larger percentage of the clinical group with score 0 and a lower percentage with score 1 and 2. However, only the differences in the distribution of title 1 scores were significant (p≤0.05), with differences in title 2 being only marginal (p=0.055).

## Discussion

Quantitative differences were found in the communicative performance between the clinical and control groups in half of the tasks in the Brazilian MAC Battery, encompassing three types of communicative processing: discursive, lexical-semantic and prosodic processing. The differences in tasks assessing discursive and prosodic components were generally more relevant than those observed in tasks evaluating the lexical-semantic component. The effect of RHB lesions on tasks examining conversational discourse abilities has been widely reported in the literature, with the following discursive characteristics: reduction of visual contact and facial expression, difficulty in choosing words to express feelings,^27^ unclear and ambiguous expression of ideas and references,^28^ reduction of vocal intonation and use of a monotonous pattern,^27^ difficulty in understanding the interlocutor’s intention and pragmatic aspects in general,^29^ among other features.

Regarding prosodic impairment, the reduction of perception and production of intonation curves of linguistic and emotional prosody has frequently been described in RHB damage conditions.^30,31^ Besides the significant differences observed between the groups in discursive and prosodic processing, RHBD individuals have also presented lower performance in verbal fluency tasks investigating lexical-semantic abilities, compared to non-brain-damaged participants. Less significant differences in lexical-semantic abilities had been expected, since this type of processing is generally more impaired in left-brain-damaged individuals.^32^

Thus, the initial hypothesis, which predicted the occurrence of significant differences between the groups for the four types of communicative processing examined by the Brazilian MAC Battery, with the least significant differences expected in the lexical-semantic component compared to the other types of processing, was not fully confirmed. A right-brain-damage effect was not found in this group study on tasks that formally examined pragmatic-inferential processing. The groups do not differ in relation to pragmatic-inferential performance in the metaphor interpretation task nor in the indirect speech acts interpretation task. Two factors may have contributed to the absence of a significant difference between the groups. The first relates to the formality of these tasks, which might have been facilitative considering the complexity generated by numerous communicative clues present in the individuals’ daily routine. This facilitation occurs specifically in RHBD individuals, who are unable to take advantage of clues from the real communicative context. This explanation is postulated due to a dissociation observed in this study between the absence of difference in metaphoric and speech acts comprehension, normal tasks, along with the presence of difference in the conversational discourse task, a functional subtest. This test is characterized by the existence of a context of real communicative exchanges between interlocutors, in which various pragmatic aspects, such as non-literal message comprehension given by the interlocutor (examiner) are evaluated to give the final score.^1^ In the literature, impaired comprehension of non-literal information, that is, of inferential processing at the conversational discursive level, is associated with RHB damage.^18,33^ The second factor consists of the probable heterogeneity present in the RHBD participants evaluated in this study, where there may be some individuals with RHB lesion in the sample who present communicative impairment and others who do not.

The heterogeneity factor is strongly correlated to the second hypothesis of this study – the clinical group will present a less homogeneous performance than the control group. This hypothesis was confirmed through observation of a pattern of higher variability among RHBD individuals compared to non-brain-damaged participants. Considering that about 50% of individuals who present an RHB vascular lesion show communicative changes and that, in the present study, the recruitment of the clinical group was not based on the inclusion criterion of the presence of communicative deficit, only 50% of this group presenting communicative deficits was expected. The notion that a lesion to the right side of the brain does not automatically impair communicative abilities was confirmed.^22^

The great variability found in the clinical group may be related to selection of an appropriate clinical group, which represents a challenge for the researcher, in order to study the effect of RHB lesion in communication.^34^ There are disadvantages in studying individuals who are undergoing rehabilitation for altered communicative patterns because they represent a distorted sample and therefore this was an exclusion criterion in the present study. On the other hand, there also are advantages of selecting an RHB damaged sample based only on the presence of the lesion per se, which was the case in this study. This type of selection allows the investigation of clinical subgroups, despite the fact that the mean of the group may confound an existing communicative deficit.

Several factors are pointed to in the literature as being responsible for the inter-subject variability verified in the RHBD samples of group studies. Briefly, this variability is linked to the inclusion of individuals with different neurological characteristics, such as lesions in different sites, with different extents, different levels of clinical severity, besides various non-neurological attributes, such as schooling, age, manual dominance, pre-morbid knowledge and abilities. Moreover, the variability in physiological and psychological adaptation that occurs in distinct periods over time and with distinct compensations for each individual may also play a role.^5,6,34,35^

In this investigation, some of these attributes have been controlled for, such as schooling, age, manual dominance, among others. However, due to the great difficulty in forming a clinical group with rigorous control over various inclusion criteria, important variables have not been totally controlled for, such as time following lesion onset and extent of vascular accidents. It is noteworthy that the implementation of this ideal *a priori* control is not possible in group studies, since it implies a methodological change in the study design: from group studies to multiple-case studies. Taking into consideration the difficulties in conducting group studies with ideal control of inter- and intra-subject variables, as well as the inherent heterogeneity of the clinical population of RHBD individuals, the development of studies of clinical profile grouping (cluster) and of individual and multiple-case studies for the investigation of dissociations has been recommended in the literature.^3,18,22^ In studies on aphasia – partial loss of language presented by the majority of left-brain-damaged individuals – heterogeneity has been reduced by grouping different clinical subgroups, which has led to the classification of aphasia typology.

Therefore, this study has identified RHB lesion effects using the Brazilian MAC Battery subtests which evaluate discursive, prosodic and lexical-semantic communicative processing, providing evidence of a more prominent effect in the two first types of processing than in the third. RHB lesion also influenced pragmatic processing in the conversational discourse task. Moreover, the RHBD group exhibited less homogeneous communicative performance compared to the control group.

In order to better understand the heterogeneity present in communicative deficits and in their manifestations following an RHB neurological problem, more empirical studies should be encouraged employing the highest methodological rigor possible. The current study, representing a pioneering study into right-brain-damaged populations in the Brazilian context, contributes with initial and illustrative results. Further studies are necessary mainly involving exploratory group investigations on the existence of different clinical profiles and case studies, including a general neuropsychological description of this neurological population.

## APPENDIX A

**Description of Brazilian MAC Battery’s tasks**

**Questionnaire on deficit awareness.** This task was administered only to the clinical sample group, since it was specifically designed for assessing awareness of communication impairments in individuals who suffer lesions along with its impact on their routines, that is, of the occurrence of anosognosia. The questionnaire is composed of seven closed questions demanding yes/no answers.

**Conversational discourse.** The examiner introduces two different themes during a ten-minute-long dialog. Four themes are suggested: family, work, leisure and current events such as the Presidential campaign. Various linguistic components are observed a posteriori through the analysis of 17 aspects (such as indifference to comments like funny remarks).

**Metaphor interpretation.** The stimuli are 20 metaphoric sentences: the 10 first ones are non-conventional metaphors in Brazilian Portuguese (e.g., *“Meu pai é um pavão” – “My father is a peacock”*) – and the 10 final ones, idiomatic expressions (e.g., *“Tenho que pôr a mão na massa” – meaning “to get stuck in” – “I have to start doing something”*). The examinee is oriented to explain the meaning of each sentence.

**Unconstrained verbal fluency.** The clinician asks the participant to say the largest number of words they can in a period of two minutes and thirty seconds.

**Linguistic prosody comprehension.** The individual is asked to identify if the intonation of simple sentences is affirmative, interrogative or imperative (e.g., *“João toma café” – “John drinks coffee”*), previously audio recorded. There are 12 sentences.

**Linguistic prosody repetition.** The participant is asked to repeat each sentence with the same intonation that was identified. The 12 stimuli are the same as the previous subtest.

**Narrative discourse.** This task, based on an oral five-paragraph narrative, presents three subtests: 1) partial retelling, paragraph by paragraph; 2) full retelling; and 3) 12 comprehension questions. The individual under examination is also asked to give the story two titles: title 1 – before the comprehension questions – and title 2 – after the comprehension questions. At the end of the task, the examiner judges whether the expected inference had been made or not, based on the participant’s answers.

**Verbal fluency with orthographic constraint.** The participant is oriented to say the largest number of words possible starting with the letter P in two minutes.

**Emotional prosody comprehension.** The examinee has to identify the intonation – happiness, sadness or anger – in 12 sentences of simple grammatical structure with a neutral content (e.g., *“Tiago vai sair” - Tiago is going to leave”*), previously audio recorded.

**Emotional prosody repetition.** The individual repeats each sentence in the same intonation that had been identified (same 12 stimuli used in the previous task).

**Indirect speech act interpretations.** The stimuli are 20 brief situations – 10 ending with a direct speech act in which the interlocutor’s intention is explicit (e.g., *“Esta nova televisão funciona muito bem” – “This new television works very well”*, meaning *“Esta nova televisão é boa” – “This new television is good”*) and the other 10 ending with an indirect speech act, in which the interlocutor’s intention is implicit (e.g., *“João, a porta do seu quarto está aberta” – “João, the door of your room is open”*, meaning *“João, feche a porta” – “João, close the door”*). The participant is asked to explain what the person intended to say.

**Verbal fluency with semantic constraint.** The examinee says the largest number of words as possible that denote pieces of clothing in two minutes.

**Emotional prosody production.** The participant is oriented to say a sentence with the intonation which expresses the emotion – happiness, sadness or anger – induced by a situation (e.g., *“Acabei de vir do médico” – “I’ve just arrived from the doctor”*). There are nine stimuli.

**Semantic judgement.** The person is initially asked to say whether there is a relationship or not between two words (*yes / no answer*); if yes, they should proceed in explaining what the relationship. A total score of the identified relationships (identification score) is calculated, as well as a total score of explanations for the existing relationships (explanation score). The stimuli are 24 word pairs, 12 composed of words with a category relationship (e.g., *maçã-ameixa / apple-plum*).
